# Life course perspective for improving oral health: strategies and interventions to integrate oral health care and primary health care in community health centers

**DOI:** 10.3389/froh.2025.1618354

**Published:** 2025-09-26

**Authors:** Mary E. Northridge, Martin Lieberman

**Affiliations:** 1 Hansjörg Wyss Department of Plastic Surgery, New York University (NYU) Grossman School of Medicine, New York, NY, United States; 2 NYU Langone Dental Medicine Postdoctoral Residency Programs, NYU Langone Hospitals, New York, NY, United States; 3 Department of Dental Medicine, Family Health Centers at NYU Langone, Brooklyn, NY, United States

**Keywords:** community health centers, dental care, dental clinics, health workforce, oral health, quality of life, training support

## Abstract

In the United States, disparities in access to quality oral health care exist at every stage across the life course. The net result is a greater likelihood of poor oral health at every age for people who live in underserved and rural communities than for people who live in communities with better access to quality oral health care. Both universal and targeted interventions at multiple levels of influence across the life course and intergenerationally are needed to eliminate disparities in access to oral health care and end the disgrace of poor oral health as the US national symbol of social inequality. While community health centers hold promise for delivering patient-centered, value-based care, they experience challenges related to the oral health literacy of patients and organizations and to the building of sufficient capacity to meet the high demand for oral health care services. To address the training needs of the US dentistry workforce, the long-term goal of the New York University Langone Dental Medicine Postdoctoral Residency Programs is to improve oral health care access and delivery across the life course for people of all ages and intergenerationally. The short-term goal is to recruit and train dentists to lead patient-centered models of integrated care delivery at community health centers in underserved and rural communities of 30 US states, Puerto Rico, and the US Virgin Islands. This paper presents the capstone findings of a 5-year postdoctoral dental residency training project built upon a foundation of shared decision-making and motivational interviewing training for dental faculty and residents. Improving patient experience and patient-reported outcomes are critical in transforming dentistry from a fee-for-service to a value-based health care model. Scaling up promising interventions and addressing time and resource constraints in community health centers require the broad commitment of communities, organizations, patients and their families in demanding and realizing the US societal goal of oral health for all.

## 1 Introduction

Several constructively critical accounts have documented the consequences of the separate systems of dental and medical care provision in the United States on disparities in access to even basic services for impoverished and underserved population groups compared with their more socioeconomically advantaged counterparts (1–5). The unmet need for quality oral health care in disadvantaged and rural populations served by community health centers (CHCs) is longstanding and considerable (6). Oral diseases and craniofacial injuries are at elevated rates throughout the life course in historically marginalized people due to determinants at multiple levels (1). Risk factors for poor oral health include but are not limited to: (1) lack of eligibility and/or high out-of-pocket costs for public and private dental insurance coverage at the societal/policy level; (2) poor access to affordable and nutritious food at the community/neighborhood level; and (3) low uptake of parental consent for preventive oral health care for their children, including vaccination against *human papillomavirus* (HPV), at the family/friends level. These multi-level determinants of oral health may affect the oral microbiota, salivary function, and teeth and their supporting structures over the life course. If left untreated and unprotected, these risk factors may lead to dental caries and facial trauma (beginning in early childhood), oropharyngeal cancer (an epidemic among US men), gingivitis and periodontal disease (more prevalent with increasing age), oral cancer (especially in older men), and eventually root caries and tooth loss (notably in older adults) (1).

A body of scientific evidence indicates that oral diseases (dental caries, periodontal diseases) and general health conditions (obesity, diabetes) are closely linked by sharing common risk factors (excess sugar consumption, tobacco use), and underlying infection and inflammatory pathways (7, 8). This project sought to: (1) integrate oral health care and primary health care at CHCs through interprofessional education (IPE) of dental faculty and residents (9, 10); (2) incorporate interventions at multiple levels to improve access to and quality of services (1); and (3) incorporate shared decision-making (SDM) and motivational interviewing (MI) techniques as essential components of patient-centered, value-based oral health care (11). The overarching goal of implementing this set of strategies and interventions was to reduce disparities in oral health across the life course.

## 2 Pedagogical framework underlying the educational and training activities

The life course approach is the study of long-term effects on chronic disease risk of physical and social exposures during gestation, childhood, adolescence, young adulthood, and later adult life (12). It aids in understanding human development throughout the lifespan and intergenerationally, with specific relevance to the study of health trajectories (13). The major oral diseases (dental caries, periodontal diseases, and oral cancers) are all relatively prevalent in the US population and largely preventable, while also exhibiting socioeconomic disparities for both clinical and self-reported oral health outcomes at all stages across the life course (14). If left untreated, oral conditions may harm oral health-related quality of life and general well-being given their impacts on dental pain, loss of functioning, and concerns regarding appearance, especially for marginalized populations facing discrimination and geographic barriers who are unable to access quality oral health care (1).

**Figure 1:**
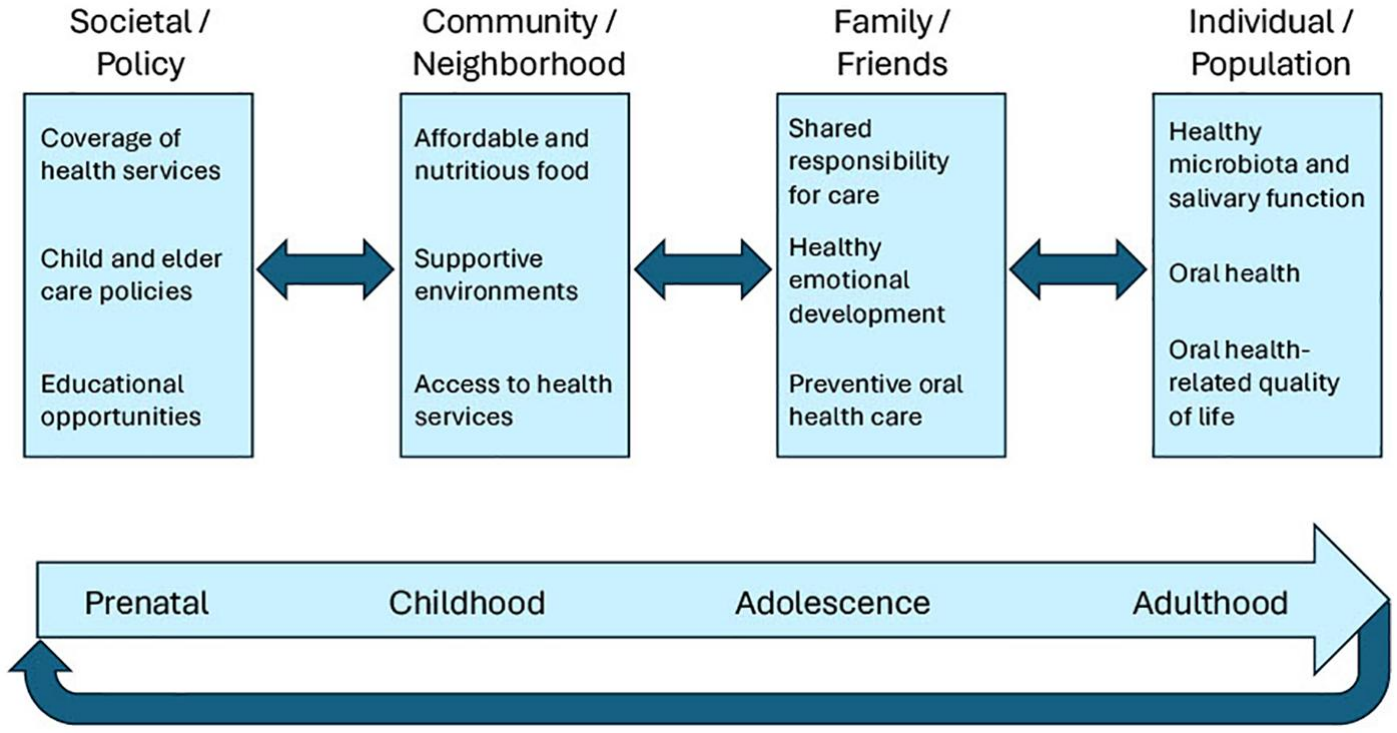
Actionable multi-level framework that incorporates the life course perspective titled, intergenerational pathways to oral health.

Inherent in the life course approach is the notion that people live in interdependence and determinants at various levels of situated networks of human relationships influence health and health care (13, 15). Figure 1 belongs to a class of social ecological models that are in widespread use (15–17) and which evolved from ongoing research and practice initiatives led by the authors. Specifically, this multi-level framework posits that factors at the *societal/policy*, *community/neighborhood*, and *family/friends* levels influence health outcomes including **healthy microbiota and salivary function**, **oral health**, and **oral health-related quality of life** at the *individual*/*population* level.

Note that at the bottom of Figure 1 is a simplified life course graphic that may be interpreted as the “circle of life”, signifying that the oral health of the mother may affect the oral health of the developing fetus. Moreover, as children, adolescents, and adults develop and age, they are influenced by the social determinants of health (SDOH) at the *societal/policy* level, e.g., **coverage of health services**, **child and elder care policies**, and **educational opportunities**. Points of intervention also exist at the *community/neighborhood* level via **affordable and nutritious food**, **supportive environments**, and **access to health services**, as well as at the *family/friends* level via **shared responsibility for care**, **healthy emotional development**, and **preventive oral health care**. This actionable multi-level framework for the project is titled, ***Intergenerational pathways to oral health***. A review led by the first author is currently under development that includes the theoretical basis for this evidence-based framework (M.E.N., personal communication).

## 3 Learning environment, learning objectives, and pedagogical format

### 3.1 Learning environment

The life course perspective was used as an organizing framework for a series of value-based care interventions to improve oral health service delivery conducted over 5 years by New York University (NYU) Langone Dental Medicine Postdoctoral Residency Programs (hereafter NYU Langone Dental Medicine). For nearly 4 decades, NYU Langone Dental Medicine program leaders, dental faculty, and staff built a network of more than 110 CHCs originating at Family Health Centers at NYU Langone, a Federally Qualified Health Center (FQHC) network in southwest Brooklyn, NY, and encompassing community-based, ambulatory care training sites located in 30 US states, Puerto Rico, and the US Virgin Islands. The mission of the 5 programs, namely, Advanced Education in General Dentistry (AEGD), Advanced Education in Pediatric Dentistry (AEPD), General Practice Residency (GPR), Dental Anesthesiology, and Endodontics, is to serve, teach, and discover (10). The 3 largest programs (AEGD, AEPD, and GPR) are considered primary care dentistry programs and are the focus of this report.

While CHCs are uniquely positioned to provide integrated, patient-centered care, they experience challenges related to both personal and organizational oral health literacy (18) and the building of sufficient capacity to meet the high demand for oral health care services (19). As per Bates et al., the decision of a dental school graduate to pursue postgraduate dental training is heavily influenced by having a residency requirement in the US state where the dentist elects to practice, as is currently mandated in New York, as well as the amount of federal graduate medical education (GME) investment in resident support (20). In addition, community-based vs. dental school-based postgraduate training increases the likelihood that dentists will participate in the Medicaid program (21) and work at CHCs (22). Even as there are other determining factors, foreign-trained dentists are more likely to participate in the Medicaid program than are US-trained dental school graduates and Black women who are dentists are more likely to work at CHCs than their peers, leading Mertz et al. to conclude that dentist workforce diversity is critical to closing the gaps in oral health care access and delivery for underserved populations (22). Finally, younger dentists are more likely to be female, members of underrepresented racial/ethnic minorities, practice in groups rather than solo, and be affiliated with dental support organizations vs. independent practices than late-career dentists, representing a major generational transition that is still underway in dentistry (23).

### 3.2 Learning objectives

The specific, measurable learning objectives of the 5-year postdoctoral training project were to:

A. Train general and pediatric dental faculty and residents to use SDM (24–26) and MI (27–29) techniques to deliver age-appropriate oral, primary, and behavioral health care services
B. Plan and test 4 interprofessional pilot projects regarding the SDOH, HPV vaccination, telehealth, and sugary drink consumption
C. Enhance the didactic and clinical IPE training of dental residents to care for patients with developmental disabilities, substance use disorders, and mental health conditions
D. Support the opportunity for dental residents to earn an *Advanced Certificate in Public Health* (ACPH) with an option for a master's in public health (MPH) degree
E. Hire NYU Langone Dental Medicine graduates to fill community-based clinical positions to expand the general and pediatric dentistry workforce

### 3.3 Pedagogical format

The following collection of 5 categories of interventions were conducted at various levels across targeted stages of the life course to enhance the IPE and training of NYU Langone Dental Medicine faculty and residents (please see Figure 1). Note that certain of these activities were briefly described in a recently published IPE use case (10). Since interventions that address factors at multiple levels may be more effective than those that involve a single level (30), whenever possible IPE and training activities targeted as many levels of influence as time and resources allowed. For instance, at the *societal/policy* level, Medicaid coverage of health services and GME funding of dental residents at CHCs are critical to implementing and sustaining each of the following types of interventions. To further illustrate the multi-level approach, at the *community/neighborhood* level, the screening and referral pilot projects sought to improve access to and coordination of care across services within CHCs, while screening dental patients/parents for the SDOH and referring them when indicated and desired to, e.g., food pantries and English as a second language classes, were designed to enhance linkages between CHCs and community resources (31). Interventions targeting the *family/friends* level were thought to be especially effective given how essential and influential the people we love and care for are in our lives, including by providing assistance with toothbrushing for young children and older adults with disabilities and cognitive impairment, actively listening to the concerns of parents/guardians regarding HPV vaccination for their children as trusted family members and confidants, and suggesting actionable strategies prioritized by young people themselves for limiting sugary drinks in their diets to promote both their oral and general health. Finally, identifying key oral health outcomes at the *individual/population* level for all age groups and suitable metrics that can be tracked over time and place is an urgent priority in moving dentistry from fee-for-service to value-based oral health care (32).

#### 3.3.1 Train faculty and residents to use SDM and MI techniques

Evidence-based approaches such as SDM and MI are effective at fostering the essential skills to build nonjudgmental provider—patient partnerships and motivate behavioral change based on the life circumstances of patients that shape their priorities (24, 27). Over the course of 5 years, both in-person and virtual foundational, intermediate, and train-the-trainer workshops, booster sessions, and one-on-one coaching and fidelity monitoring sessions were developed with MI experts and institutional partners and iteratively enhanced by the interdisciplinary project team for NYU Langone Dental Medicine faculty and residents. In the final 2 years of the project, remote objective structured clinical exercise training sessions delivered via the Zoom platform (ZOSCEs) were conducted with faculty and residents where standardized participants (SPs) previously trained as patients rotated between 3 different breakout rooms so that faculty and residents were able to participate in all 3 developed scenarios: (1) screening and referral for the SDOH with an older adult dental patient; (2) parental consideration of the HPV vaccine for a 9-year-old dental patient; and (3) engagement with an obese adolescent dental patient to decrease their sugary drink intake. Feedback from SPs (patient actors) was critical in better ensuring the developed scenarios were tailored to providing patient-centered care and provider—patient engagement (33).

#### 3.3.2 Plan and test interprofessional pilot projects

Using Family Health Centers at NYU Langone as a “learning laboratory” and collaborating across services, 4 interprofessional pilot projects were planned and tested to improve oral health across the life course, as listed in Table 1.

**Table 1:** Interprofessional pilot projects conducted at Family Health Centers at NYU Langone in Brooklyn, New York, USA to improve oral health across the life course and useful references.

Interprofessional pilot project	Population targeted	Useful references^a^
Screening and referral for the social determinants of health	Parents/guardians of pediatric dental patients; behavioral health patients	(31, 33)
Screening and referral for *human papillomavirus* vaccination	Parents/guardians of pediatric dental patients	(33, 34)
Screening and referral for oral health care using a telehealth platform	Behavioral health patients at intake, both in-person and virtual	(33, 35)
“Rethink Your Drink” educational campaign to improve oral and general health	Pediatric and adolescent dental patients and their families/friends	(33), Supplementary file

a All internally and externally published references are available from the corresponding author upon request.

Note that preliminary findings were reported for the intervention regarding the SDOH (31) as well as the rationale for dentists screening and referring patients aged 9–15 years for HPV vaccination (34).

For the third intervention that was funded in response to the *coronavirus disease 2019* (COVID-19) pandemic, the project team was able to leverage the successful telehealth platform of the Behavioral Health Program and screen all patients at intake for time of and reason for last dental visit, with patient referral for oral health care if requested (35). The following year, enhanced referral and follow-up across NYU Langone Health services became possible given the implementation of an integrated (medical, dental, behavioral health, and social services) electronic health record (EHR) known as Epic with Wisdom.

Finally, a culturally competent sugary beverage educational campaign (“Rethink Your Drink”) to reach diverse, low health literacy patients and their families was adapted, implemented, and evaluated by GPR program faculty and residents, with quantitative and qualitative results presented at 2 consecutive NYU Langone Dental Medicine Annual Research Fairs held in June 2022 and June 2023. Note that one of the developed posters was included in a SDM and MI training scenario for dental faculty and residents to engage with an obese adolescent dental patient actor to decrease their sugary drink intake (33).

#### 3.3.3 Enhance IPE course offerings

NYU Langone Dental Medicine utilizes the online learning management system titled, *Brightspace*. Working with an experienced and talented instructional designer hired for the project to ensure optimal hosting and delivery of the online programming, content experts across institutions and disciplines created IPE courses with modules on developmental disabilities, the SDOH, the opioid epidemic, mental health, sugary drinks, and tele-dentistry to complement the clinical training of residents at CHCs. As innovations in technology, science, adult education, and online platforms become available through the extensive NYU Langone Health operations and resources, the courses and their constituent modules are updated and refined by the content experts who serve as course instructors and the NYU Langone Dental Medicine *Graduate Dental Education* (GDE) team members, with input from the requisite evaluations completed by learners.

#### 3.3.4 Support the opportunity to earn an ACPH

NYU School of Global Public Health partnered with NYU Langone Dental Medicine and the Health Resources and Services Administration (HRSA) to offer scholarships to dental residents to earn an ACPH designed to enhance knowledge and training in core public health concepts (epidemiology, social and behavioral health, biostatistics, environmental health, health care policy, and public health management and leadership). Delivery of the program through an innovative online format provides residents the opportunity to complete the certificate while training and participate in a vibrant online community with public health faculty and fellow students. The first 2 cohorts of enrolled residents were offered 3 years to complete the ACPH program, whereas the second 2 cohorts of enrolled residents were offered 2 years to complete the ACPH program.

#### 3.3.5 Hire NYU Langone dental medicine graduates as dental faculty at CHCs

The high cost of dental education and consequent loan burdens contribute to the shortage of dentists who work at CHCs and serve as faculty members (36). To fill community-based clinical positions and expand the dental faculty workforce, NYU Langone Dental Medicine is committed to training its residents in IPE and integrated models of care delivery and hiring its graduates.

## 4 Results to date

Major societal and organizational changes occurred over the 5-year project period, including dental faculty furloughs and program staff turnovers that were accelerated by the COVID-19 pandemic, greater scientific understanding of the effectiveness of the HPV vaccine in preventing cancers along with increased vaccine hesitancy in certain population groups, and rapid technological advances that facilitated remote education and training of dental faculty and residents, even as many people experienced isolation and lack of social connectedness during this time period. A rigorous and tailored application of the Plan-Do-Study-Act (PDSA) cycle approach, as recommended for establishing genuine learning organizations (37), was used for each of the QI interventions, and adaptations across CHCs and refinements of workflows remain in process. Below we report key experiences, outcomes, and aftermaths for each of the 6 categories of interventions that were conducted to enrich the professional skills and hiring and promotion prospects of the involved faculty and residents.

### 4.1 SDM and MI training

Of all the interventions, the SDM and MI training underwent the most radical upheaval of its workplan (33) yet ultimately proved to be the most transformative for NYU Langone Dental Medicine. The positive dental faculty experience with the ZOSCE learning format delivered by an accessible and responsive team of New York Simulation Center for the Health Sciences (NYSIM) hosts, staff, and SPs (patient actors) is summarized in the following participant quotation. “*I'd like to reiterate how impressive the training session was yesterday. While the subject matter was top-notch [I always learn something new from (MI consultant)], I really do think that's the ﬁrst [continuing education] course I've been a part of that has used the potential of Zoom effectively. The training was actively enhanced [using] the Zoom features. This really is the ﬁrst time I've taken a virtual learning course where I felt that it did things that wouldn't have been possible with an in person learning experience. It was exciting to be a part of the future of learning*”.

Moreover, AEPD program faculty and residents are tailoring and implementing QI projects regarding screening and referral of dental patients for the SDOH and HPV vaccination in their own CHCs based upon adaptations of the first and second patient scenarios for the ZOSCE sessions. Meanwhile, AEGD program faculty and residents conducted a group patient education project in the last academic year regarding sugary drink intake based on the third ZOSCE scenario and presented their results at the NYU Langone Dental Medicine Annual Research Fair held in June 2024. Finally, the AEGD program faculty and residents are finalizing a group patient education project on HPV vaccination in the current academic year inspired by the second ZOSCE scenario to be presented at the forthcoming Research Fair to be held in June 2025. All these extension initiatives of the project are intentionally scaling up the IPE and integrated practice priorities of NYU Langone Dental Medicine, fulfilling Commission on Dental Accreditation (CODA) standards for residents to engage in scholarly activity or clinical research (38), and providing structured research opportunities for faculty and residents that are team-based and geared toward improving patient experience and patient-reported outcomes as part of value-based oral health care (11).

### 4.2 Interprofessional pilot projects

While the referral process at Family Health Centers at NYU Langone is more seamless since the implementation of the integrated EHR on November 1, 2021, organizational changes, workflow refinements, and re-education of providers and staff across departments are also needed to fully realize the benefits of patient-centered care in an accessible location (30). As an illustrative example, SmartSet clinical decision support now alerts a medical provider at wellness visits when a patient has not visited a dentist in 18 months or longer, effectively doubling the number of referrals to dentistry and orthodontics from ∼500 referrals per month to ∼1,000 referrals per month (39). This notable success nonetheless required adjusting the staffing model to provide outreach to ∼1,000 patients per month and oral health care to markedly more patients, especially children (39). Thus, the scale-up of new workflows needs to be phased in.

In support of these integration efforts, the project team developed targeted ZOSCE training scenarios regarding interprofessional care toward fostering confidence in faculty and residents to build nonjudgmental dentist—patient partnerships (33). The collaboration between the Department of Dental Medicine and the Behavioral Health Program has been especially rewarding since the workflow is now fully institutionalized, with behavioral health providers screening all of their patients at intake (both in-person and virtual) for oral health and health care needs and the SDOH, with on-site referral for dental care upon patient request at the Sunset Terrace location. Since the behavioral health patients who present for oral health care are at elevated need of treatment services (35), this partnership helps to restore dignity, health, and well-being to an often stigmatized and marginalized patient population.

In terms of the culturally competent sugary beverage educational campaign, in Spring 2022 a short, written survey was developed by the GPR faculty and residents and completed by their patients at a dental clinic in Sunset Park, Brooklyn to help identify useful strategies to educate and modify the sugary drink intake of their culturally diverse, socioeconomically disadvantaged patient population. Results included that a large percentage of patient respondents did not believe that New York City tap water is safe to drink. This finding was then incorporated into one of the “Rethink Your Drink” educational posters displayed in the dental clinics (please see the Supplementary file for the presentation titled, “Rethink Your Drink”).

Finally, in Spring 2023 a focus group was conducted by the GPR faculty and residents with 11 adolescents from Project Reach Youth regarding oral health, visiting the dentist, and beverages that they and their friends and family drink. The focus group participants also provided valued feedback on the “Rethink Your Drink” campaign and helped select a more universally applicable poster to support dental faculty and residents in engaging with an obese adolescent dental patient regarding their sugary drink intake during the ZOSCE training sessions (33). The scenario used to train the SPs (patient actors) contains the excerpt provided below. Note that it includes a possible discussion aided by the aforementioned “Rethink Your Drink” poster (33) as part of the explore-ask-explore MI approach, wherein the dentist first asks the SP about their knowledge of sugary drinks before offering any unsolicited advice.

If the dentist asks: “Would it be alright if I shared some information with you about how sugary drinks affect your oral health?”

[SP] answers: “Sure—I guess”.

If the dentist responds: “Here is a poster that shows how much sugar there is in a supersized energy drink and a large soda. What do you think about that?”

[SP] responds: “That is huge! I never realized how much sugar is packed into a single bottle or can.”

[SP] expresses astonishment at the information conveyed in the poster and talks freely with the dentist about how unaware they were that soft drink companies add large amounts of sugar to what they and their friends drink every day.

### 4.3 Enhanced *Brightspace* courses

In partnership with the NYU School of Global Public Health and expert dental consultants, NYU Langone Dental Medicine developed the following 3 courses available to all faculty and residents on its *Brightspace* platform in the initial 2 years of the project, with ongoing review by staff and learners and further enhancements planned as resources and time allow.

(1) Public Health and Dentistry
  - Module 1: Mental and Oral Health: A Public Health Perspective
  - Module 2: Substance Use and Dental Health: A Public Health Perspective
  - Module 3: Sugary Beverages and Public Health Dentistry
  - Module 4: Social Determinants of Oral Health: Science and Clinical Implications
(2) Dental Care for Adults with Intellectual and Developmental Disabilities (IDDs)
  Part I: Dental Care for Adults with IDDs
    - Module 1: Introduction
    - Modules 2–5: Dental Care for People with Down Syndrome, Cerebral Palsy, Fragile X, Fetal Alcohol Syndrome, and Autism Spectrum Disorder
    - Module 6: Medicolegal Considerations
    - Modules 7 and 8: Treatment Planning (2 parts)
    - Module 9: Behavioral Management
  Part II: Motivational Interviewing
  Part III: Working with People with Various Special Needs
(3) Teledentistry
  - Module 1: Teledentistry: Overview
  - Module 2: Implementation of Teledentistry
  - Module 3: Case Studies and Applications of Teledentistry

### 4.4 ACPH program graduates

As of Spring 2025, 17 dental residents were graduates of the ACPH program, even as there are still scores of dental residents who were accepted by their program directors and may yet receive their certificates. The Vice President of Graduate Dental Education (M.L.) considers this number of graduates to be impressive, given the start-up administrative challenges exacerbated by the COVID-19 pandemic and the hectic clinical training environments at CHCs. If future opportunities present themselves to offer scholarships to earn an ACPH for dental residents, a more selective process will be used to ensure that applicants plan to manage oral health programs, evaluate systems of care, and design surveillance systems to measure oral health status upon graduation.

### 4.5 Graduates hired as dental faculty at CHCs

In the final analysis, this project was successful only if NYU Langone Dental Medicine was able to recruit and train dentists to lead patient-centered models of integrated care delivery at CHCs in underserved and rural areas of 30 US states, Puerto Rico, and the US Virgin Islands. Table 2 provides the numbers of graduates hired since 2018 among active dental faculty in Spring 2025, by program and overall.

**Table 2:** Graduates of the 5 NYU Langone dental medicine postdoctoral residency programs hired since 2018 among active dental faculty in spring 2025, by program and overall.

Program	Number graduates hired since 2018	Number active dental faculty in spring 2025	Percent graduates among active faculty
Advanced Education in General Dentistry (AEGD)	82	730	11.2%
Advanced Education in Pediatric Dentistry (AEPD)	24	148	16.2%
Dental Anesthesiology	4	30	13.3%
Endodontics	1	12	8.3%
General Practice Residency (GPR)^a^	3	30	10.0%
Total	114	950	12.0%

a GPR graduates are expected to practice for 5 years before they supervise residents as faculty.

Of 950 active dental faculty in Spring 2025, 114 (12%) were graduates hired since 2018. To place these numbers in context, HRSA estimates that of the more than 200,000 active dentists in the United States in 2023, only 5,167 were employed in CHCs (40). A total of 370 AEGD, AEPD, and GPR trainees each year provide oral health care to CHC patients, with 908 active dental faculty practicing and supervising them in these 3 programs alone (please see Table 2). This sums to 1,278 primary care dentists working at CHCs either employed by NYU Langone Dental Medicine or its CHC partners. In other words, 1,278/5,167 = 24.7% or nearly one-quarter of the US dentist workforce at CHCs is part of the NYU Langone Dental Medicine network. Note that this percentage does not include graduates who work at CHCs but are not active dental faculty of NYU Langone Dental Medicine.

In other data compiled by program staff for a competing continuation of the project (40), 32% of the total current AEGD, AEPD, and GPR residents are from a rural background, a disadvantaged background, or an underrepresented minority with no resident counted more than once, as per the program definitions in the notice of funding opportunity (40). Finally, 47% of AEGD, AEPD, and GPR program completers over the past 2 academic years currently practice in health professional shortage areas (41).

## 5 Discussion on the practical implications

The findings presented here of 5 categories of interventions to improve oral health care delivery across the life course are not especially novel in and of themselves. Taken together, however, they are mutually reinforcing in supporting dentists who work at CHCs to move from a fee-for-service to a value-based health care (VBHC) model that prioritizes improving patient experiences and outcomes while reducing costs to health care systems (42). The historical and continued separation of the oral health care delivery system from the medical care delivery system in the United States (1–5) has contributed to the siloed innovation culture in dentistry that has been evolving separately from that of the broader medical system. While opportunities and challenges to implementing oral health VBHC have been debated (43), CHCs are already moving forward with integrated models of care delivery, and oral health care is essential to include in these demonstration projects since oral health is vital to general health and well-being (1, 9, 14).

Strengths of this project include the large number of participating dental faculty and residents across several programs from multiple US states and territories and numerous CHCs, as well as the expertise of talented partners involved in creating the content and delivering the interventions. Limitations include the administrative burden involved in working across multiple cost centers within and between institutions and the poor connectivity for online IPE and ZOSCE trainings at certain CHCs. We hope to alleviate in part the technology difficulties experienced through supplemental funds from HRSA to purchase laptops and tablets for faculty and resident use.

According to the Beryl Institute, human experience in health care is grounded in the experiences of patients and families, members of the health care workforce, and the communities they serve, and defines patient experience as, “The sum of all interactions, shaped by an organization's culture, that influence patient perceptions across the continuum of care” (44). Patient-reported outcomes are defined by the US government as, “Any report coming directly from patients (i.e., study subjects) about a health condition and its treatment” (45) To better equip NYU Langone Dental Medicine faculty and residents with the skills to be successful in integrated care settings, SDM and MI training with patient actors and peers providing immediate feedback on interprofessional care scenarios was key. This training will form the basis for the next phase of the project, when the project team and its partners plan to leverage recent advancements in automated data collection as follows. The day after their dental appointment, CHC patients will receive clinic-branded communication in their preferred format (short message service, e-mail) in any of 75 self-selected languages requesting feedback about their experiences and self-reported oral health outcomes. Patient feedback will then be aggregated and displayed in a custom dashboard for administrators, dental providers, and staff to highlight successes and target areas for improvement. The generated machine learning ready data will thus produce actionable, targeted insights to help dental teams improve value across 14 patient experience metrics, 4 patient-reported outcome metrics, and a net promoter score. Both federal agency and private foundation funding proposals have been submitted by NYU Langone Health leaders for financial support of the planned follow-up initiative to better ensure sustained momentum and continued success.

## 6 Acknowledgment of constraints

The COVID-19 pandemic led to major impacts on the 5-year postdoctoral training project, notably the cancellation of in-person faculty development conferences for the first 3 years, the loss of institutional and external partners to conduct SDM and MI training activities, and the trauma and grief experienced by patients, providers, and staff due to tremendous loss and chronic stress that are still reverberating through health care systems and communities alike. To the extent possible, the work plan was adapted to account for these challenges by recruiting new partners and leveraging the talents of program staff. New technologies were also incorporated as they emerged, notably integrated EHRs, interactive virtual teaching and training platforms, and artificial intelligence to better support dental faculty and residents in delivering patient-centered, value-based care.

Nonetheless, the severe time and resource constraints at CHCs need to be acknowledged. Due to pressing patient emergencies and the shortage of dentists at partner sites, faculty and residents were not always available to attend planned IPE offerings and ZOSCE trainings. Hence, NYU Langone Dental Medicine remains committed to training dental residents in patient-centered, value-based care at CHCs in 30 US states, Puerto Rico, and the US Virgin Islands. If graduates retain their commitment to working in rural and underserved communities and possess the skills needed to thrive in safety net settings, these preliminary success stories will be scaled up to ever more CHCs throughout the United States.

## Data Availability

The raw data supporting the conclusions of this article will be made available by the authors, without undue reservation.
